# Skin Microbiome, Nanotoxicology, and Regulatory Gaps: Chronic Cosmetic Exposure and Skin Barrier Dysfunction—A Systematic Review

**DOI:** 10.3390/pharmaceutics17101246

**Published:** 2025-09-24

**Authors:** Loredana-Elena Pîrvulescu, Sorana-Cristiana Popescu, Roman Popescu, Vlad-Mihai Voiculescu, Carolina Negrei

**Affiliations:** 1 Department of Toxicology, Faculty of Pharmacy, “Carol Davila” University of Medicine and Pharmacy, 020945 Bucharest, Romania; elena-loredana.niculae@drd.umfcd.ro (L.-E.P.); sorana-cristiana.pruna@drd.umfcd.ro (S.-C.P.); carolina.negrei@umfcd.ro (C.N.); 2 Department of Orthopaedics, “Carol Davila” University of Medicine and Pharmacy, 020021 Bucharest, Romania; popescu_roman@yahoo.com; 3 Department of Dermato-oncology, “Carol Davila” University of Medicine and Pharmacy, 050474 Bucharest, Romania; 4 Elias University Emergency Hospital–Marasti Boulevard, No.17, District 1, 011461 Bucharest, Romania

**Keywords:** nanoparticles, skin barrier, microbiome, gut–skin axis, nanotoxicology, chronic exposure, regulatory gaps

## Abstract

**Background:** Engineered nanoparticles (NPs)—titanium dioxide, silver, zinc oxide and silica—are widely used in cosmetics for UV protection, antimicrobial activity and texturising effects. Chronic consumer-level exposure may impair skin-barrier integrity, disturb microbiome composition and dysregulate immune signalling via the gut–skin axis. Current regulatory frameworks typically omit chronic- or microbiome-focused safety assessments, leaving potential gaps. **Objectives:** This study aimed to evaluate the long-term effects of cosmetic-relevant NPs (titanium dioxide, silver, zinc oxide, silica) on skin and gut microbiota, epithelial-barrier integrity and immune signalling—including telocyte- and exosome-mediated pathways—and to identify regulatory shortcomings, particularly the absence of microbiome endpoints, validated chronic models and consideration of vulnerable populations. **Methods:** Following PRISMA 2020, PubMed, Scopus and Web of Science were searched for English-language in vivo animal or human studies (December 2014–April 2025) meeting chronic-exposure criteria (≥90 days in rodents or >10% of lifespan in other species; for humans, prolonged, repetitive application over months to years consistent with cosmetic use). Although not registered in PROSPERO, the review adhered to a pre-specified protocol. Two independent reviewers screened studies; risk of bias was assessed using a modified SYRCLE tool (animal) or adapted NIH guidance (zebrafish). Owing to heterogeneity, findings were synthesised narratively. **Results:** Of 600 records, 450 unique articles were screened, 50 full texts were assessed and 12 studies were included. Oral exposure predominated and was associated with dysbiosis, barrier impairment, immune modulation and metabolic effects. Dermal models showed outcomes from minimal change to pronounced immune activation, contingent on host susceptibility. Comparative human–animal findings are summarised; telocyte and exosome pathways were largely unexplored. Regulatory reviews (EU SCCS, US FDA and selected Asian frameworks) revealed no requirements for chronic microbiome endpoints. **Limitations:** Evidence is limited by the small number of eligible studies, heterogeneity in NP characteristics and exposure routes, predominance of animal models and a scarcity of longitudinal human data. **Conclusions:** Cosmetic nanoparticles may disrupt the microbiome, compromise barrier integrity and trigger immune dysregulation—risks amplified in vulnerable users. Existing regulations lack requirements for chronic exposure, microbiome endpoints and testing in vulnerable groups, and neglect mechanistic pathways involving telocytes and exosomes. Long-term, real-world exposure studies integrating gut–skin microbiome and immune outcomes, and harmonised global nanomaterial-safety standards, are needed to ensure safer cosmetic innovation.

## 1. Introduction

The skin is the body’s largest organ, functioning as a complex, multi-layered barrier that separates internal systems from the external environment. It comprises three distinct layers with specialised roles: the epidermis, primarily formed of stratified squamous epithelium and keratinocytes; the dermis, consisting of dense, irregular connective tissue rich in structural proteins and immune cells; and the hypodermis, a subcutaneous layer composed of adipose and loose connective tissue. Beyond its physical protective role, the skin is essential for immune defence, neuroendocrine communication, microbial balance, and detoxification processes, making it a dynamic organ in maintaining overall homeostasis [1].

The epidermis plays a central role in maintaining the skin’s permeability barrier. Keratinocytes differentiate into a layer of dead corneocytes embedded in lipid bilayers, forming a protective shield [2]. Langerhans cells act as antigen-presenting cells, mediating activation and tolerance [3], while antimicrobial peptides contribute to barrier defence and help shape microbial balance [4]. Beneath, the dermis provides structural support through collagen and elastin fibres and contains immune cells such as mast cells, dendritic cells, and macrophages, which contribute to immunological defence. The dermis also contains hair follicles and sebaceous glands, which may serve as entry points for microorganisms and nanoparticles. The hypodermis, composed primarily of adipose tissue, functions in mechanical cushioning and thermoregulation and contributes to immune regulation through adipokine secretion [4]

The skin serves as a critical immunological gatekeeper, with resident immune cells responding to xenobiotics and nanoparticles by activating pro-inflammatory pathways, particularly when barrier integrity is compromised [5]. Because the skin is continuously exposed to topical substances, it is crucial to evaluate percutaneous absorption, including the potential for long-term accumulation of nanoparticles. Appendageal structures such as hair follicles and sebaceous glands, as well as sites of compromised barrier function, can enable deeper dermal penetration of nanomaterials. Once deposited, these materials may alter local immune activity and, in some contexts, pose a risk of systemic effects [6,7].Beyond local effects, nanoparticles engage systemic stress and inflammatory pathways implicated in neurotoxicity [8].

Building on this overview of skin anatomy and physiology, it is essential to explore the factors that contribute to barrier dysfunction and microbial dysbiosis. Environmental and behavioural factors—including frequent exposure to chemical agents in cosmetics—can compromise the barrier and disrupt microbial balance. Ambient particulate matter, polycyclic aromatic hydrocarbons, and ozone induce oxidative stress, disturb barrier lipids and tight junctions, and increase transepidermal water loss; nitrogen dioxide is also recognised among key air pollutants implicated in extrinsic skin ageing and inflammation [9,10]. Surfactants are associated with keratinocyte injury, barrier damage, and pH disturbance [11]. Triclosan has been shown to disrupt keratinocyte function and skin integrity in reconstructed human epidermis [12]. These disruptions may modulate immune responses and could increase the skin’s susceptibility to nanoparticle penetration [11,12].

Nanoparticles used in topical products—such as TiO_2_/ZnO and silver—can provoke oxidative and immune responses in skin, particularly with repeated exposure or occupational contact [13,14]. In parallel, lifestyle factors, including diet quality, adequate sleep, and stress management, influence sebum production, cytokine profiles, and cutaneous immunity [15].

The gut–skin axis (Table 1) refers to the bidirectional relationship between the gut microbiota, systemic immune responses, and skin homeostasis. Recent evidence indicates that gut dysbiosis—an imbalance in intestinal microbial populations—can negatively affect skin health. It is associated with impaired barrier function and the exacerbation of inflammatory skin diseases such as acne, atopic dermatitis, psoriasis, and rosacea [16]. One mechanism involves increased intestinal permeability (“leaky gut”), permitting lipopolysaccharides and other microbe-derived products to enter the bloodstream. These circulating endotoxins engage pattern-recognition receptors, including Toll-like receptors, and skew Th1 and Th17 pathways. The resulting systemic inflammation disrupts skin-barrier repair and promotes cutaneous immune activation [17]. Short-chain fatty acids (SCFAs)—including butyrate, propionate, and acetate—produced by a balanced gut microbiota regulate epithelial proliferation, support barrier function, and promote anti-inflammatory signalling; reductions in SCFAs can contribute to dysregulated host responses [18].

Beyond SCFAs, postbiotics—non-viable microbial products and metabolites—are reported to support barrier repair, modulate pH and immune signalling, and are being explored as dermatological interventions [19]. The gut also communicates with the skin through the hypothalamic–pituitary–adrenal (HPA) axis; this neuroendocrine pathway modulates cortisol and systemic cytokine tone, with downstream effects on inflammation and barrier physiology [20].

**Figure 1:**
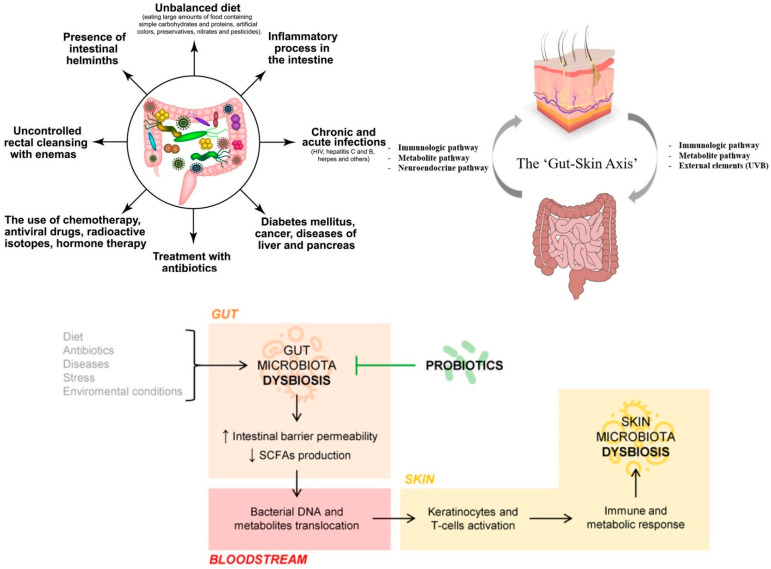
Dysbiosis: potential causes and putative mechanisms (Created in https://BioRender.com (accessed on 30 June 2025). Popescu-Sorana Cristiana, 2025).

Dysbiosis (Figure 1), whether in the gut or on the skin, may enhance host susceptibility to the toxicological effects of nanomaterials. This is particularly relevant for chronic, low-dose exposure via topical cosmetics, ingestion, or inhalation [21]. In line with EPA/IRIS, chronic exposure denotes repeated oral, dermal, or inhalation exposure for more than ~10% of a human lifespan (roughly >90 days to 2 years in commonly used laboratory species); note that many test guidelines refer to ~90-day rodent studies as subchronic [22].

Dysbiosis—whether in the gut or on the skin—may increase susceptibility to the toxicological effects of nanoparticles by weakening epithelial homeostasis. In the intestine, inorganic nanoparticles can disrupt tight junctions and cross the epithelium via paracellular routes or M-cell uptake, with downstream immune activation and gut–liver axis involvement; such barrier effects and microbiome shifts are linked to inflammatory conditions. On the skin, dysbiosis is associated with impaired barrier repair and heightened cutaneous inflammation within the gut–skin axis framework. Together, these observations suggest that microbiota composition and barrier integrity modulate nanoparticle toxicity across epithelial surfaces [23,24].

Nanoparticles such as zinc oxide (ZnO) and titanium dioxide (TiO_2_) are used in sunscreens for UV protection. Silver nanoparticles are explored for antimicrobial functions [25]. ZnO nanoparticles also show antifungal activity against *Microsporumcanis* in veterinary isolates [26]. Despite these benefits, concerns persist regarding long-term safety with repeated dermal use or inadvertent ingestion; potential risks discussed in the nanotoxicology literature include barrier interactions, microbiome perturbation, and immune dysregulation. Regulatory guidance has been updated (EU SCCS 2023; US FDA), but important data gaps remain, underscoring the need for nano-specific, chronic safety assessments for consumers and workers [27,28].

Recent studies emphasise the role of stromal cellular networks—particularly **telocytes**—in maintaining epithelial integrity and immune homeostasis in the skin and gut. Telocytes are interstitial cells with long telopodes that enable intercellular communication, stromal–epithelial signalling, and support for tissue repair and regeneration [29,30]. They occupy key microenvironments, including the dermis and the intestinal lamina propria, where they interface with immune and stem cells and the extracellular matrix [29,30]. Chronic nanoparticle exposure may perturb stromal signalling via oxidative stress and inflammation and could secondarily affect telocyte networks; however, direct telocyte-specific evidence is currently limited, and this mechanism is inferred from established nanoparticle effects in skin and gut tissues [16,21].

Telocytes and exosomes are integral components of the skin and gut microenvironment, regulating epithelial repair, immune balance, and microbiome–host communication [29,30,31]. Exosomes—nanometre-sized extracellular vesicles released by epithelial and immune cells—are critical mediators of intercellular communication. They transport bioactive molecules, including proteins, lipids, and microRNAs (miRNAs), which regulate immune responses, epithelial repair, and host–microbiome interactions [31].

In nanotoxicology, chronic exposure to engineered nanomaterials can modulate extracellular vesicle biogenesis, secretion, cargo, and uptake, linking particle exposure to altered cell–cell signalling and inflammation [32]. Sustained oxidative and inflammatory stress from nanoparticle exposure may also perturb stromal signalling and, by extension, telocyte–epithelial crosstalk; however, telocyte-specific data remain limited and require dedicated study [29,30,32]. Incorporating exosome endpoints—and developing telocyte-relevant readouts—into chronic nanoparticle testing would sharpen mechanistic understanding and support systems-biology-based safety frameworks [32].

**Figure 2:**
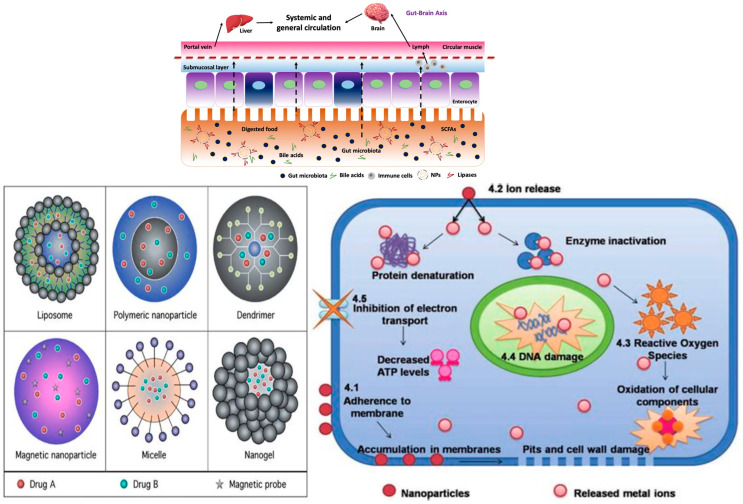
Nanoparticles and cellular mechanisms of signal disruption (Created in https://BioRender.com (accessed on 30 June 2025). Popescu-Sorana Cristiana, 2025).

**Figure 3:**
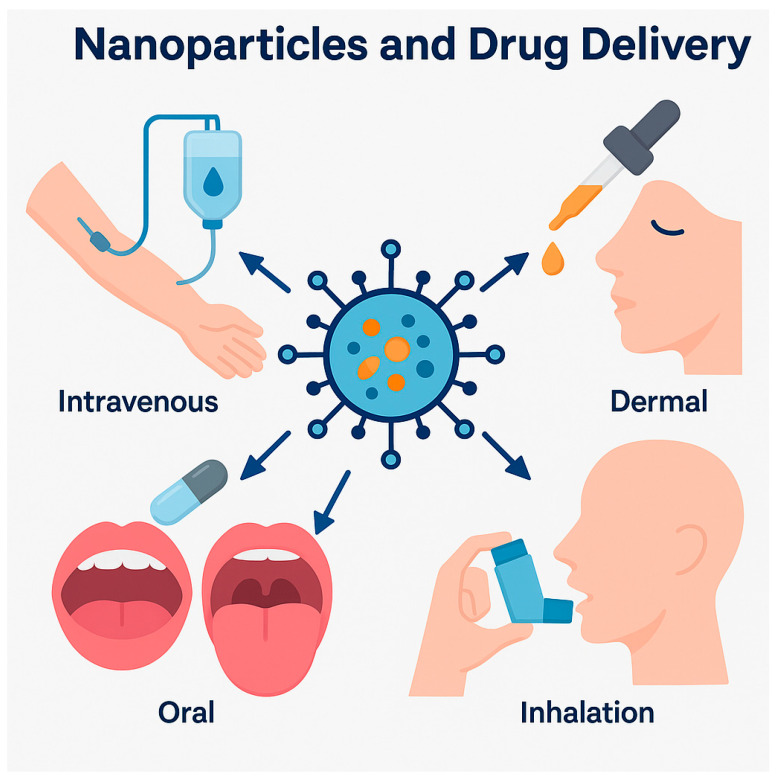
Nanoparticles and drug delivery.

Nanoparticles (Figure 2 and Figure 3) can disrupt the microbiome by exerting direct antimicrobial effects and indirectly modulating mucosal immune responses. In experimental models, common cosmetic nanoparticles—silver, zinc oxide, and titanium dioxide—alter gut microbial composition, often reducing *Lactobacillus* and *Bifidobacterium* and, in some contexts, enriching pathobionts; these changes associate with increased intestinal permeability and elevated pro-inflammatory cytokines (e.g., IL-6, TNF-α) [33,34,35]. Individuals with pre-existing dysbiosis or barrier compromise (e.g., after antibiotics, stress, diet, or pollution) may be particularly susceptible within the gut–skin axis framework [36]. Oral exposure to food-relevant nanoparticles can shift short-chain-fatty-acid-producing taxa and raise systemic cytokines; such inflammation is implicated in epithelial-barrier dysfunction relevant to skin disease [33,34,37].

Most current safety assessments of nanomaterials overlook critical factors such as host microbiome status (healthy versus dysbiotic), cumulative exposures across the gut–skin axis, and the longitudinal effects on microbial resilience and recovery. Although a substantial literature exists on nanoparticle safety, most toxicological assessments focus on acute or subchronic exposures, physicochemical characterisation, or single-organ outcomes. Systematic evaluations of chronic, consumer-relevant exposure that integrate microbiome endpoints, telocyte/exosome pathways, and gut–skin axis interactions remain scarce. Current regulatory frameworks (e.g., EU SCCS, US FDA) do not mandate chronic, microbiome-integrated testing for cosmetic nanomaterials, leaving a critical gap in consumer protection [27,28].

We emphasise the urgent need for in vivo longitudinal studies to assess the cumulative and systemic effects of nanoparticle exposure, especially in individuals with altered microbiota or weakened epithelial barriers. Understanding these chronic interactions is essential for developing microbiome-conscious risk assessments, sustainable cosmetic formulations, and evidence-based regulatory policies that better protect consumer health.

This systematic review evaluates the chronic effects of cosmetic-relevant engineered nanoparticles (silver, titanium dioxide, zinc oxide, silica) on skin and gut microbiota, epithelial barrier integrity, and immune signalling—including telocyte- and exosome-mediated pathways—and identifies regulatory gaps in current safety assessments, with particular emphasis on the absence of microbiome-integrated endpoints, validated chronic-exposure models, and consideration of vulnerable populations.

## 2. Materials and Methods

**Protocol and Registration:** While the protocol was not prospectively registered in PROSPERO or other registries, it was developed prior to data collection and followed PRISMA 2020 (Preferred Reporting Items for Systematic Reviews and Meta-Analyses) guidelines to ensure methodological transparency and reproducibility [38]. Because the review’s scope focuses on mechanistic toxicology, regulatory analysis, and microbiome-related outcomes, it falls outside the primary clinical-intervention focus of most registration platforms. A comprehensive protocol outlining the objectives, eligibility criteria, search strategy, and planned synthesis methods was developed in advance and is available upon request.

**Methods Section Objective (PICO):**

- **Population (P):** Healthy and susceptible populations in in vivo animal or human studies, including immunocompromised, pregnant, and metabolically vulnerable models.
- **Intervention/Exposure (I):** Chronic or repeated exposure to engineered nanoparticles used in cosmetics (silver, titanium dioxide, zinc oxide, silica) via dermal or oral routes relevant to consumer use. Chronic exposure was defined according to U.S. EPA criteria as repeated exposure exceeding 10% of the organism’s lifespan (>90 days in rodents), applied to both dermal and oral routes [22].
- **Comparator (C):** Unexposed controls or alternative formulations (e.g., bulk materials, ionic forms, conventional cosmetics without nanoparticles).
- **Outcomes (O):**
  ○ Skin and gut microbiota composition and diversity
  ○ Epithelial barrier integrity (cutaneous and intestinal)
  ○ Immune signalling pathways, with emphasis on telocyte- and exosome-mediated communication
  ○ Adverse health effects relevant to long-term cosmetic use
- **Additional objective:** Identify and discuss gaps in regulatory frameworks—particularly the absence of microbiome-integrated toxicity endpoints and validated chronic-exposure models—and propose systems-biology approaches to improve safety assessments.

**Search Strategy:**

A systematic literature search was performed on 15 April 2025 across PubMed (NCBI), Scopus (Elsevier), and Web of Science Core Collection (Clarivate). Searches targeted peer-reviewed articles in English from 1 December 2014 to 15 April 2025. Strategies combined MeSH/controlled terms with free text related to nanoparticles, microbiota, skin barrier integrity, chronic exposure, and regulatory assessment.

The primary search terms included:

- **“Nanoparticles AND skin barrier”**
- **“Nanoparticles AND gut microbiota”**
- **“Chronic exposure AND cosmetics”**
- **“Nanotoxicology AND regulation”**

**PubMed Boolean String**

((“Nanoparticles”[MeSH Terms] ORnanoparticles[Title/Abstract]) AND

((“Skin Barrier”[MeSH Terms] OR“skin barrier”[Title/Abstract]) OR (“Gastrointestinal Microbiome”[MeSH Terms] OR“gut microbiota”[Title/Abstract]) OR (“chronic exposure”[Title/Abstract] AND (cosmetics[MeSH Terms] ORcosmetics[Title/Abstract]))

OR (nanotoxicology[Title/Abstract] AND (regulation[Title/Abstract] ORlegislation[Title/Abstract] ORpolicy[Title/Abstract])))) AND (“12 January 2014”[Date-Publication]:“15 April 2025”[Date-Publication])AND (English[lang])

**Scopus Boolean String**

(TITLE-ABS-KEY(nanoparticles) AND (TITLE-ABS-KEY(“skin barrier”)

OR TITLE-ABS-KEY(“gut microbiota”) OR (TITLE-ABS-KEY(“chronic exposure”) AND TITLE-ABS-KEY(cosmetics)) OR (TITLE-ABS-KEY(nanotoxicology) AND (TITLE-ABS-KEY(regulation) OR TITLE-ABS-KEY(legislation) OR TITLE-ABS-KEY(policy)))))AND PUBYEAR > 2014 AND PUBYEAR < 2026 AND (LIMIT-TO(LANGUAGE, “English”))(TITLE-ABS-KEY(nanoparticles) AND (TITLE-ABS-KEY(“skin barrier”) OR TITLE-ABS-KEY(“gut microbiota”) OR (TITLE-ABS-KEY(“chronic exposure”) AND TITLE-ABS-KEY(cosmetics)) OR (TITLE-ABS-KEY(nanotoxicology) AND (TITLE-ABS-KEY(regulation) OR TI TLE-ABS-KEY(legislation) OR TITLE-ABS-KEY(policy)))))AND PUBYEAR > 2013 AND PUBYEAR < 2026 AND (LIMIT-TO(LANGUAGE, “English”))

**Web of Science Boolean String**

TS = (nanoparticles AND (“skin barrier” OR “gut microbiota” OR (“chronic exposure” AND cosmetics)

OR (nanotoxicology AND (regulation OR legislation OR policy))))

AND PY = 2014–2025

AND LA = (English)

Search strategies were adapted to each database’s syntax and indexing. Reference lists of eligible studies and relevant reviews were screened to identify additional records.

**Inclusion and Exclusion Criteria**

Studies were eligible if they met all the following:

1. Publication type and date: Peer-reviewed original research articles or systematic reviews published between 1 December 2014 and 15 April 2025.
2. Study design and population: In vivo animal or human studies, involving healthy or susceptible populations (e.g., immunocompromised, pregnant, metabolically vulnerable models).
3. Exposure: Chronic or repeated exposure—via dermal or oral routes—to cosmetic-relevant engineered nanoparticles (e.g., silver, titanium dioxide, zinc oxide, silica), including realistic consumer-use or occupational scenarios.
  ○ **Chronic exposure definition:** “Repeated exposure by the oral, dermal, or inhalation route for more than approximately 10% of the life span in humans (more than approximately 90 days to 2 years in typically used laboratory animal species)” [22].
4. Outcomes: At least one of the following:
  ○ Skin or gut microbiota composition/diversity
  ○ Epithelial barrier integrity (cutaneous or intestinal)
  ○ Immune signalling pathways, with emphasis on telocyte and/or exosome-mediated communication
  ○ Regulatory or safety assessment aspects related to chronic nanoparticle exposure
5. Relevance to synthesis groups:
  ○ Studies assessing biological outcomes (microbiota, barrier, immune markers) were included in the mechanistic toxicology synthesis.
  ○ Studies addressing regulatory context or safety frameworks were included in the regulatory gap analysis synthesis.
  ○ Studies meeting both criteria contributed to both syntheses.

Studies were excluded if they met any of the following:

1. In vitro-only experiments, non-peer-reviewed content, or publications before 2014.
2. Focus exclusively on physicochemical nanoparticle characterisation without biological or regulatory endpoints.
3. Non-English publications.
4. Studies unrelated to skin, gut, microbiota, or immune signalling (e.g., purely environmental monitoring without human/animal relevance).
5. Acute exposure models without relevance to chronic cosmetic or consumer-use scenarios.

**Data Extraction**

**Selection Process**

Two reviewers (S.-C.P. and R.P.) independently screened all records at both title/abstract and full-text stages. Disagreements were resolved through discussion, with V.-M.V. and C.N. acting as arbiters when consensus was not reached. No automation tools were used. No records required translation.

**Data Collection Process**

Two reviewers (S.-C.P. and R.P.) independently extracted data using a predefined, structured form that was piloted on a sample of studies to ensure consistency. Extracted variables included:

- Study design and model (species, health status, sample size)
- Nanoparticle characteristics (type, size, surface properties)
- Exposure route and duration
- Outcomes on microbiota (gut or skin), epithelial barrier integrity, immune markers (e.g., cytokines), and telocyte/exosome involvement
- Regulatory context, if reported
- Relevance to chronic cosmetic exposure scenarios

All time points and all measures reported for these outcomes were extracted when available. Where data was missing or unclear, no imputation was performed; original reports were checked, and authors were not contacted. No automation tools or figure-extraction software were used.

**Data Items**

Outcomes: All reported results within each outcome domain (microbiota, barrier integrity, immune markers, telocyte/exosome pathways) were extracted for all time points measured.

Other variables: Participant/animal characteristics, nanoparticle physicochemical properties, exposure details, and funding/conflict-of-interest statements (if reported) were collected. If these were not reported, they were recorded as “not stated”.

**Risk of Bias Assessment**

Although a formal risk-of-bias meta-analysis was not feasible, we prioritised well-controlled in vivo studies that used doses relevant to human exposure scenarios. All included studies were screened for methodological clarity, presence of appropriate control groups, and outcome measures aligned with the review objectives

Risk of bias was assessed independently by two reviewers using a modified version of the SYRCLE (Systematic Review Centre for Laboratory Animal Experimentation) tool, tailored for in vivo studies. Domains assessed included: random allocation, blinding of investigators, blinding of outcome assessors, completeness of outcome data, selective reporting, and other potential sources of bias (e.g., unrealistic dosing, absence of control groups). Judgements and brief textual justifications are presented in Table 2.

Risk of bias was assessed independently by two reviewers using a modified SYRCLE tool for in vivo studies. For the single zebrafish model, NIH quality assessment guidelines for non-human studies were adapted. Judgements and justifications are in Table 2.

**Effect Measures**

Given the heterogeneity of designs, no meta-analysis was conducted. Where available, extracted measures included:

- Microbial abundance and diversity metrics (e.g., *Lactobacillus* counts)
- Barrier integrity measures (e.g., transepidermal water loss)
- Immune biomarkers (e.g., IL-6, TNF-α)
- Telocyte/exosome-related parameters

No thresholds or ranges for interpreting effect sizes were applied.

**Synthesis Methods**

Studies were assigned to one or both synthesis groups (mechanistic toxicology, regulatory gap analysis) according to pre-specified eligibility criteria.

No subgroup analyses, sensitivity analyses, or data conversions were required.

**Reporting Bias Assessment**

Although no formal statistical or graphical tests (e.g., funnel plots) were feasible due to the small number of studies and heterogeneity in outcomes, we assessed the potential for reporting bias narratively. This included evaluating each study for selective outcome reporting against its stated objectives, considering missing or inconsistently reported endpoints, and cross-checking outcomes across multiple reports of the same study when available. No major discrepancies suggesting selective reporting were identified.

**Certainty Assessment**

Certainty ratings of “moderate” or “low” were assigned based on a structured qualitative framework considering: (1) study design (in vivo animal vs. human), (2) sample size adequacy, (3) consistency of effect direction across independent studies, (4) directness of the exposure scenario to real-world cosmetic use, and (5) overall risk of bias judgments. These criteria, while not a formal GRADE process, align with its core domains of risk of bias, inconsistency, indirectness, imprecision, and publication bias.

**Figure 4:**
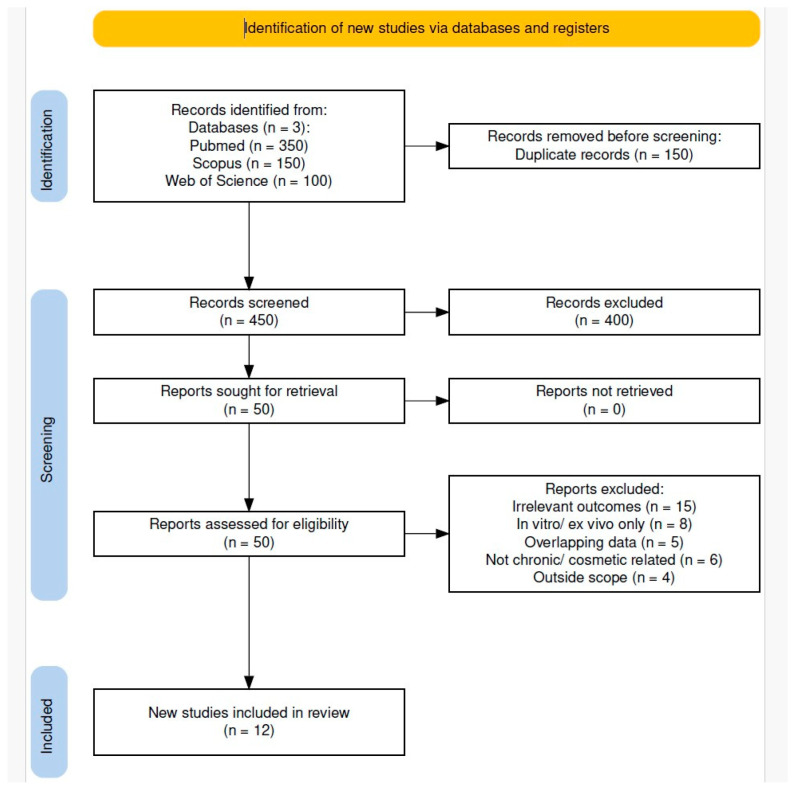
PRISMA 2020 flow diagram of study selection.

As shown in Figure 4, the database search initially retrieved 600 records. After removing 150 duplicates, 450 unique articles remained for screening. Of these, 400 were excluded based on title and abstract review. Most exclusions were due to off-topic content or in vitro-only studies.

Fifty full-text articles were assessed for eligibility. Thirty-eight were excluded based on predefined criteria, resulting in twelve studies that met all inclusion criteria and were included in the qualitative synthesis.

**Reasons for Exclusion:**

Following a detailed full-text eligibility assessment, studies were excluded for the following reasons:

1. Lack of relevant outcomes—Fifteen studies did not investigate skin barrier function and gut microbiota alterations concerning cosmetic nanoparticles [51,52,53,54,55,56,57,58,59,60,61,62,63,64,65].
2. In vitro or ex vivo only—Eight studies relied exclusively on in vitro or ex vivo models without in vivo data or physiological relevance [66,67,68,69,70,71,72,73].
3. Duplicate or overlapping data—Five articles presented duplicate or overlapping findings, such as short communications reiterating results from full research papers, or narrative reviews offering no additional original data [74,75,76,77,78].
4. Not chronic/cosmetic related—Six studies focused on acute, high-dose exposures, not representative of cosmetic or consumer use scenarios [79,80,81,82,83,84].
5. Outside scope—Four studies examined only environmental effects or physicochemical properties of nanoparticles without addressing health-related endpoints [85,86,87,88,89].

**Quality Assessment:**

Each included study was evaluated for the relevance of its dosing regimen to real-world human exposure scenarios. Priority was given to studies using doses reflective of typical consumer use rather than artificially high concentrations. Data were extracted directly as reported; therefore, statistical conversions, imputations, or standardisation procedures were not required. Due to the limited number of eligible studies and substantial heterogeneity in design, endpoints, and exposure conditions, sensitivity analyses and formal assessments of heterogeneity (e.g., subgroup analysis or meta-regression) were not feasible.

**Certainty of Evidence**

The overall certainty of evidence was rated as moderate to low. A formal GRADE evaluation was not undertaken; however, confidence in the findings was reduced by small sample sizes, variable outcome measures, and lack of human studies.

Consistent patterns across independent in vivo experiments strengthen the plausibility of the observed microbiome and immune effects.

## 3. Results

**Study Selection and Characteristics**

Following the multi-database search and screening process (Figure 4), twelve studies met the inclusion criteria. Silica nanoparticles (SiNPs) were the most common overall among included studies [39,41,48,49,50], while titanium dioxide (TiO_2_) and silver nanoparticles (AgNPs) predominated among oral-exposure studies [42,43,44,45,46,47]. Across studies, AgNPs produced effects ranging from minimal to pronounced, and TiO_2_ also consistently altered gut microbiota and metabolism; therefore, the comparative magnitude of diversity loss between AgNPs and TiO_2_ was mixed within this dataset (Table 3). Dermal exposures were less common and ranged from negligible systemic effects to immune sensitisation, with outcomes influenced by host susceptibility. Zinc oxide (ZnO) studies highlighted haematological, biochemical, and organ-specific toxicities in ingestion models [40]. Collectively, outcomes varied by nanoparticle type, host physiology, and exposure route (Table 3).

**Exposure types and models**

Oral exposure was the most common route, simulating dietary or ingestion-based contact. TiO_2_ was examined in zebrafish and in healthy or pregnant rats [42,46,47], with reported gut-microbiota alterations, metabolic changes, inflammation, and increased maternal blood glucose (gestational exposure). AgNPs in mice showed mixed outcomes: negligible change over 28 days in one study [43], microbiota disturbance without histopathology in another [44], and long-term dysbiosis with neurobehavioural changes after developmental exposure [45]. ZnO, compared with zinc salts, in mice produced distinct haematological and organ-specific toxicities [40]. In rats, nanoparticles, including SiO_2_ and AgNPs, were associated with subclinical microbiome and metabolome shifts without overt toxicity [41] [clarified: portfolio of NPs rather than co-administration]. In immunodeficient mice, oral SiO_2_ altered gut microbiota without systemic toxicity, underscoring immune status as a determinant of NP effects [48].

Dermal exposure was assessed in three studies. SiNPs applied to rats for 90 days produced no systemic toxicity at tested doses [39]. SiNP agglomerates co-applied with allergen in mice elicited an IgE-skewed response and increased anaphylaxis sensitivity [49]. In a murine allergic contact dermatitis model, amorphous SiO_2_ NPs reduced inflammation, indicating context-dependent immunomodulation [50].

**Sample Sizes and Population Types**

Most studies used mice [40,43,44,45,48,49,50], rats [39,41,46,47], and one zebrafish model [42]. Models included healthy animals, immunodeficient mice [48], and pregnant rats [47].

**Health Status and Observed Effects**

Responses varied with nanoparticle type, dose, and host condition. Gut-microbiota dysbiosis was common with TiO_2_ [42,46,47] and observed with AgNPs in several contexts [44,45], while ZnO produced haematological and histopathological toxicity [40]. Developmental exposures [45,47] were associated with persistent microbial, metabolic, and behavioural effects. Dermal exposures ranged from no systemic toxicity [39] to immune sensitisation [49] or reduced inflammation [50]. In immunodeficient mice, SiO_2_ altered microbiota without systemic effects [48].

**Main Outcomes**

Minimal impact: Ryu et al. [39], Wilding et al. [43], Palmer et al. [50] reported negligible or context-dependent beneficial changes.

Significant impact: Yazdanshenas et al. [40], Landsiedel et al. [41], Chen et al. [42], and Mao et al. [47] observed microbiota disruption, metabolic changes, or immune modulation.

Developmental/metabolic risks: Lyu et al. [45] and Mao et al. [47] linked perinatal/pregnancy exposures to persistent metabolic and behavioural changes.

Immunological risks: Shabbir et al. [48] demonstrated heightened susceptibility in immunocompromised hosts; Hirai et al. [49] showed allergen co-exposure increases hypersensitivity.

To contextualise these biological mechanistic outcomes within a broader safety perspective, Table 4 aligns animal-model findings with existing human evidence for each nanoparticle type, while identifying regulatory gaps that persist in chronic cosmetic exposure assessments.

## 4. Discussion

**Research Gap**

Although research on nanoparticle toxicology is expanding, systematic evaluations of chronic, low-dose NP exposure on the skin–gut axis remain scarce. Few studies use microbiome-specific endpoints or examine mechanistic pathways involving telocytes and exosome-mediated communication—both critical for barrier homeostasis.

Only a minority of included studies assessed both gut and skin outcomes directly. However, several reported systemic immune and metabolic changes after oral NP exposure, suggesting downstream cutaneous effects. This supports a biologically plausible gut–skin axis interaction, even if direct causal links were not measured in all cases.

**Effects of nanoparticles on gut microbiota and the intestinal barrier**

Across the twelve included studies, TiO_2_ and AgNPs were the most frequently investigated overall; TiO_2_ and AgNPs predominated among oral exposures, whereas SiO_2_ featured most in dermal models (see Table 3). Gut microbiota disruption and epithelial-barrier impairment were common findings. ZnO and SiO_2_ NPs were less studied but also showed measurable microbiota shifts or immune modulation. Oral exposure dominated the study designs. Dermal exposure studies were fewer, but results ranged from negligible immune effects to pronounced immune activation, suggesting route-specific hazard profiles.

A review by Utembe et al. [89] confirms that metallic and metal-oxide NPs can influence gut microbial ecosystems. Oral and indirect exposures commonly led to gut dysbiosis, metabolic disruption, and immune modulation—findings consistent with those for TiO_2_, AgNPs, ZnO and SiO_2_ in our synthesis.

Rinninella et al. [90] report that dietary TiO_2_ exposure alters gut-microbiota composition and is associated with impaired barrier indices (e.g., tight-junction/mucus changes) across several animal models; the direction of change for specific taxa—including Akkermansiamuciniphila—varies by study. These observations align with Chen et al. [42,46], who found TiO_2_-related microbiota shifts accompanied by oxidative stress and intestinal inflammation.

Bettini et al. [91] extended these observations to a potential carcinogenic risk: in rats, food-grade TiO2 (E171) impaired immune homeostasis, initiated preneoplastic lesions, and promoted aberrant crypt development. Human carcinogenicity data remain limited and are under regulatory review.

Mohammed et al. [92] and Khabir et al. [93] reported minimal cutaneous absorption of ZnO NPs in healthy human volunteers. However, these findings apply to topical use and do not rule out the oral or inhalation toxicity seen in rodent ingestion and workplace-exposure models [40].

Babaei et al. [94] reported elevated inflammatory cytokine expression in industrial workers chronically exposed to silver and silica nanoparticles. This supports animal data [43,44] showing the immunostimulatory potential of NPs and suggests that subclinical inflammation seen in vivo may have real-world relevance.

Ratanapokasatit et al. [95] demonstrated that the skin microbiome contributes to the ageing process, emphasising links between commensal diversity, skin integrity, and immune defence.

Naik et al. [96] provided foundational evidence that resident skin commensals compartmentalise local immunity, inducing protective T-cell responses that maintain cutaneous homeostasis and limit hypersensitivity. By contrast, nanoparticle toxicity depends strongly on surface coatings/functionalisation and the surrounding formulation matrix; excipients such as emulsifiers and surfactants can modify nano–microbiota interactions and real-world outcomes [97,98,99,100].

**Effects of Nanoparticles on Skin Barrier and Dermal Health**

Recent advances in nanotechnology have led to widespread use of engineered nanoparticles (NPs) such as silica (SiNPs), titanium dioxide (TiO_2_) and silver (AgNPs) in cosmetics, sunscreens and topical medications. Evidence indicates that dermal exposure to these materials can disrupt the skin barrier, alter immune responses and, in some contexts, trigger systemic effects—particularly in vulnerable or sensitised individuals.

Ryu et al. [39] reported no apparent systemic toxicity after 90-day dermal SiNP exposure under the tested conditions. In contrast, Hirai et al. [49] found that co-exposure with allergens triggered an IgE-skewed immune response and increased anaphylactic sensitivity, demonstrating the adjuvant potential of SiNPs. Palmer et al. [50] observed that amorphous SiNPs modulated allergic contact dermatitis, reducing inflammation in some cases—suggesting biphasic immunomodulation depending on host status.

Yoshioka et al. [98] showed that nanomaterials can penetrate damaged or sensitised skin and trigger allergic responses via toll-like receptor activation and dendritic-cell stimulation—of particular concern in atopic dermatitis and occupational settings. Babaei et al. [94] further reported elevated inflammatory-cytokine expression in workers chronically exposed to SiNPs and AgNPs, consistent with dermal-to-systemic immune activation seen in animal models.

Regulatory oversight remains insufficient. Consistent with Henkler et al. [97] and Allan et al. [99], current cosmetic safety evaluations often rely on conventional toxicology endpoints; NP-specific assessments of barrier interactions, immune outcomes, and long-term microbiome effects are limited and uneven across jurisdictions.

While ZnO penetration was minimal in healthy volunteers [92,93], animal and in-vitro studies indicate that mechanical damage, chronic use or permeation enhancers can increase absorption, highlighting a critical knowledge and surveillance gap [101].

The gut–skin axis functions as a bidirectional regulatory network. Several oral-exposure studies [45,46,47] show that TiO_2_ and AgNPs can disrupt microbiota, induce inflammation and alter metabolism. Similar effects may occur after dermal exposure under certain conditions. Pinget et al. [102] and Cao et al. [103] confirmed TiO_2_-mediated disruption of microbiota–immune communication, while Ruiz et al. [104] linked TiO_2_ to worsened colitis via NLRP3-inflammasome activation. Ma et al. [105] detailed the mechanisms underlying TiO2-related hepatotoxicity, strengthening the connections between the gut, liver, and skin. Weir et al. [100] emphasised TiO_2_’s ubiquity in food and cosmetics, raising concerns about chronic multi-route exposure, especially in children. Chen et al. [46,106] reported TiO2-associated alterations in gut microbiota, disrupted amino acid metabolism, and liver injury—effects that may exacerbate skin inflammation when barriers are compromised. Similar microbiota disruption and metabolic changes after prenatal or early-life NP exposure were reported by Lyu et al. [45] and Mao et al. [47].

Agans et al. [107] found that TiO_2_ nanoparticles exert a lower direct inhibitory effect on human gut microbiota than silver nanoparticles, aligning with reports that oral AgNP exposure can disturb murine gut communities [44] and that silica nanoparticles can remodel gut microbiota in immunodeficient mice [48].

In paediatric populations, Zhou et al. [107] reported ZnO-associated microbiota changes in both healthy and ADHD-diagnosed children, raising neuro-immune considerations; related susceptibility has been observed in perinatal rodent models [45].

Additional studies highlight complex effects on dermal immunity. Wang et al. [108] found that ZnO induced dermal immunosuppression in a contact-hypersensitivity model. Júnior et al. [109] developed AgNP-containing dermal substitutes, showing therapeutic promise but also potential safety risks. Mukhopadhyay et al. [110] enhanced antimicrobial efficacy using peptide–NP combinations but noted toxicity concerns. Najahi-Missaoui et al. [111] stressed the absence of clearly defined safe-exposure thresholds.

Compared with gut-microbiota research, evidence on NP-induced skin-microbiota change remains limited. Existing studies focus mainly on oxidative stress and barrier integrity. High-resolution microbiome profiling is needed to clarify causal relationships. While most systemic immune and metabolic outcomes discussed here stem from oral or developmental exposures, cross-compartment immune shifts suggest that similar systemic effects could occur after intact-skin exposure.

**Immune and Inflammatory Responses**

Multiple studies show that nanoparticles (NPs) activate innate immune sensors such as the NLRP3 inflammasome, worsening inflammatory outcomes via oxidative-stress pathways, including ROS–TXNIP signalling [112]. Co-exposures can amplify these effects: for example, antibiotics combined with AgNPs intensified systemic immune changes and altered microbiota composition [113]. These patterns mirror dermal models in which silica NPs triggered hypersensitivity reactions [49,50].

Oral TiO_2_ exposure is recurrently associated with gut-microbiota disruption, elevated pro-inflammatory cytokines and hepatotoxicity in animal models, with effect size varying by dose, matrix and host status [46,47,105,114]. These findings implicate the gut–liver axis as a key mediator of systemic NP effects—especially relevant in occupational settings where dermal absorption can occur alongside ingestion [115,116].

In vitro ‘leaky-gut’ work shows that nanoparticle-based carriers can traverse compromised epithelium and deliver siRNA targeting JAK1, illustrating how nanoformulations may modulate immune signalling when barriers are impaired, without implying that unmodified nanoparticles intrinsically activate JAK1 [117].

Across NP types, AgNPs appear more harmful to microbial viability and diversity than TiO_2_ [107,118]. ZnO NPs have been shown to alter gut-microbiota composition in both healthy children and those with ADHD [107]. Together, these outcomes support gut dysbiosis as a potential sentinel biomarker for NP toxicity.

Dermal studies indicate that NP penetration and immunotoxicity vary with particle size, surface charge and formulation. Beyond direct effects, NP-induced oxidative stress and interference with redox signalling may influence cutaneous homeostasis; in combination with size, charge and formulation, these factors condition dermal penetration and immunotoxicity [119,120].

Novel transdermal systems and nanomedical applications highlight the dual nature of these materials—offering therapeutic benefits but also posing safety risks—underscoring the need for safety frameworks that evaluate both gut and skin microbiome endpoints [121,122].

Importantly, none of the included studies assessed telocyte or exosome parameters—two key components of epithelial-barrier maintenance. Telocytes coordinate stromal–epithelial communication, while exosomes carry regulatory microRNAs and proteins that guide barrier repair and immune regulation. Disruption of these systems by chronic NP exposure could link microbiome dysbiosis to sustained barrier dysfunction. This mechanistic bridge between the gut–liver–skin axis and telocyte/exosome signalling represents a critical research gap; future toxicology studies should integrate these endpoints to capture early, subclinical disruptions that current regulatory testing overlooks.

**Regulatory Implications and Gaps**

This review compared cosmetic nanomaterial regulations across major jurisdictions, including the EU Cosmetics Regulation (EC No 1223/2009), SCCS Notes of Guidance, the U.S. FDA framework, and selected Asian, Canadian, and Australian systems (Table 5).

The EU SCCS mandates acute and subchronic toxicity testing for cosmetic nanoparticles but does not require chronic exposure models or microbiome/immune endpoints. Dermal long-term NP testing is not a requirement, and pregnancy-specific risk evaluation is absent.

The U.S. FDA does not mandate pre-market approval for cosmetics (except colour additives) and has no NP-specific chronic exposure or microbiome testing requirements. Safety evaluations rely heavily on manufacturer-submitted data without independent verification, leaving transparency and consistency gaps.

In Asia (Japan, South Korea, China), regulations focus on ingredient labelling and acute dermal safety. Chronic exposure, immune, or microbiome endpoints are not addressed, and labelling requirements for NPs vary widely.

Canada uses a case-by-case approach under the Cosmetic Regulations, without a standardised NP chronic exposure framework or microbiome integration.

Australia requires notification for new industrial chemicals, including NPs, but has no specific chronic cosmetic-use or microbiome testing guidelines. Dermal penetration guidance is minimal.

Despite growing evidence of biological impacts of engineered nanoparticles (NPs) on host systems, regulatory frameworks remain fragmented and insufficient. Studies by Chen et al. [46], Mao et al. [47] and Shabbir et al. [48] report significant gut-microbiota alterations and metabolic disruptions following oral exposure to titanium-dioxide and silica nanoparticles. Yet no standardised guidelines require microbiome-specific toxicological endpoints—a critical oversight in risk assessment.

Our synthesis indicates that chronic NP exposure, even at consumer-relevant doses, can induce microbiota and immune changes that current standard toxicology assays may miss. For example, TiO_2_ and AgNPs—widely used in sunscreens and creams—are not subject to mandatory chronic dermal testing or microbiome evaluation. Silica NPs in leave-on cosmetics also lack formal safety thresholds, despite murine co-exposure studies identifying immune-sensitisation risks [44]. These omissions allow cumulative-exposure products to bypass evaluation for subtle but biologically significant effects.

Cutaneous studies [49,50] further show that silica and other NPs can have immunomodulatory and pro-allergic effects. Nonetheless, dermal risks are often underestimated or excluded from safety assessments, even amid growing evidence of occupational exposure [114,115].

The epithelial-barrier hypothesis [123,124] proposes that environmental agents, including NPs, compromise epithelial integrity, contributing to chronic conditions such as allergy, autoimmunity and inflammatory bowel disease. This concept is absent from NP regulations, and cumulative or low-dose chronic effects are rarely addressed in testing guidelines. TiO_2_ has been shown to exacerbate experimental colitis via NLRP3 pathways [106,112], and multiple studies report NP-associated dysbiosis and immune perturbation [118,125].

Vulnerable groups—such as children and immunocompromised individuals—show greater susceptibility to NP effects in developmental and immunodeficient animal models [45,47]. Current safety thresholds seldom account for these populations, limiting applicability and protection. Globally, regulation remains inconsistent; Allan et al. [99] highlight the lack of harmonisation across jurisdictions despite calls to integrate longitudinal studies, host-susceptibility factors and microbiome endpoints into hazard-identification protocols.

**Strengths and Limitations**

This review used a comprehensive multi-database search with predefined chronic-exposure criteria applicable to animal and human contexts. It integrated mechanistic, toxicological and regulatory perspectives, and included a human–animal hazard comparison to aid cross-species interpretation. However, only a small number of studies met the inclusion criteria, and most data came from animal models. Heterogeneity in NP characteristics (size, coating, charge) and outcome measures precluded meta-analysis. None of the included studies assessed telocyte or exosome endpoints, limiting mechanistic depth. Gut–skin-axis effects were inferred mainly from systemic outcomes rather than measured directly. The dataset was too small and heterogeneous for a formal publication bias assessment.

**Implications for Research and Policy**

Closing these gaps will require coordinated research and targeted regulatory reform. Future safety assessments for cosmetic nanoparticles should mandate chronic-exposure testing (≥10% of lifespan in animal models) and equivalent long-term-use scenarios in humans. Evaluations must integrate microbiome endpoints for both gut and skin to capture subtle but clinically relevant shifts. Mechanistic studies should incorporate telocyte and exosome measurements, given their roles in barrier integrity and immune regulation. Pre-market testing should address vulnerable populations (pregnant individuals, immunocompromised patients, those with metabolic disorders). Nanoparticle physicochemical characteristics (size, shape, surface charge, coating) should be reported in a standardised manner to enable cross-study comparisons. Finally, harmonising labelling and testing requirements across jurisdictions is essential to close regulatory blind spots and establish consistent global safety standards.

## 5. Conclusions

Nanoparticles (NPs), widely used in cosmetics—particularly titanium dioxide, silver, zinc oxide, and silica—can disrupt the gut and skin microbiome, impair epithelial barrier integrity, and alter immune function, with effects amplified in vulnerable populations, such as those with dermatological disorders, immunodeficiencies, or genetic susceptibility to dysbiosis.

While oral-exposure models most consistently reveal these impacts, dermal exposure also poses risks, and host condition strongly influences outcomes. Despite mounting mechanistic evidence, current regulatory frameworks lack requirements for chronic-exposure models, microbiome-integrated endpoints or testing in vulnerable populations. Critical pathways involving telocytes and exosomes, which may link dysbiosis to barrier dysfunction, remain absent from toxicological assessments.

To ensure consumer safety, regulators should mandate chronic-exposure testing that reflects real-world cosmetic use, integrate microbiome and immune endpoints, assess telocyte and exosome function, include vulnerable-population models and harmonise nanomaterial-safety requirements globally. Implementing these measures would close key regulatory gaps, align testing with emerging scientific evidence and safeguard long-term public health.

## Figures and Tables

**Table 1:** Skin–Gut Axis Overview.

Component	Function	Interaction with Microbiota	Impact of Dysbiosis
Skin Barrier	Physical and immunological protection	Supports commensal bacteria, produces antimicrobial peptides	Inflammation, increased permeability
Gut Barrier	Nutrient absorption, immune modulation	Produces short-chain fatty acids, trains the immune system	Leaky gut, systemic inflammation
Hypothalamic–Pituitary–Adrenal axis	Stress and neuroendocrine regulation	Bidirectional gut–skin signalling via cortisol	Cortisol dysregulation, immune shifts
Microbiome (Gut/Skin)	Homeostasis, pathogen resistance	Maintains immune balance, produces metabolites	Supports inflammation, immune dysregulation

**Table 2:** Risk of Bias Assessment.

Study (Author, Year)	Random Allocation	Blinding of Investigators	Blinded Outcome Assessment	Incomplete Outcome Data	Selective Reporting	Other Bias (e.g., Dose Relevance)	Overall Risk of Bias
Ryu et al. (2014) [39]	Unclear	No	No	Low	Low	Moderate	High
Yazdanshenas et al. (2025) [40]	Unclear	No	No	Low	Unclear	Moderate	High
Landsiedel et al. (2022) [41]	Low	Unclear	No	Low	Low	Low	Moderate
Chen et al. (2018) [42]	Unclear	No	No	Low	Unclear	Moderate	High
Wilding et al. (2016) [43]	Low	Low	Unclear	Low	Low	Low	Low
Van den Brule et al. (2016) [44]	Low	Unclear	Unclear	Low	Low	Low	Moderate
Lyu et al. (2021) [45]	Unclear	No	No	Low	Low	Moderate	High
Chen et al. (2019) [46]	Unclear	No	No	Low	Unclear	Moderate	High
Mao et al. (2019) [47]	Unclear	No	No	Low	Unclear	Moderate	High
Shabbir et al. (2023) [48]	Low	Unclear	No	Low	Low	Moderate	Moderate
Hirai et al. (2015) [49]	Low	Low	Unclear	Low	Low	Low	Low
Palmer et al. (2019) [50]	Low	Low	Low	Low	Low	Low	Low

**Table 3:** Characteristics and key findings of included studies.

Study	Exposure	Nanoparticle Type	Sample Size	Health Status	Model	Key Findings	Authors’ Conclusions	Regulatory Gap	Funding/Conflict of Interest
1. Ryu et al. (2014) [39]	Topical	Silica NPs	100	Healthy	Rats	No systemic toxicity after 90-day	Safe at tested dose	Limited human data	Funding—Yes Conflict of interest—No
2. Yazdanshenas et al. (2025) [40]	Oral	ZnO NPs + salts	45	Healthy	Mice	Hematologic, biochemical, and histopathologic changes; ZnCl_2_ is most toxic	ZnCl_2_ is more toxic than ZnO NPs	Lack of comparative safety guidelines	Funding—NS Conflict of interest—No
3. Landsiedel et al. (2022) [41]	Oral	SiO_2_,AgNPs	NS	Healthy	Rats	Gut microbiota and plasma metabolite shifts	Subclinical effects observed	No microbiome endpoints in safety assessments	Funding—Yes Conflict of interest—No
4. Chen et al.(2018) [42]	Oral	TiO_2_ NPs	NS	Healthy	Zebrafish	Microbiota dysbiosis, inflammation, and increased permeability	Adverse host effects	Lack of co-exposure assessments	Funding/Conflict of Interest—No
5. Wilding et al. (2016) [43]	Oral	AgNPs	5/cage	Healthy	Mice	No major microbiota change	Minimal impact	Need chronic exposure data	Funding/Conflict of Interest—NS
6. Van den Brule et al.(2016) [44]	Oral	AgNPs	6 female	Healthy	Mice	Microbiota shifts, no histopathological change	Disturbed microbiota	Missing subclinical endpoints	Funding/Conflict of Interest—No
7. Lyu et al. (2021) [45]	Oral	AgNPs	12 dams	Healthy	Mice	Offspring: dysbiosis, increased adiposity, behaviour changes	Gut–brain axis impact	No long-term neurodevelopment data	Funding—Yes Conflict of interest—No
8. Chen et al. (2019) [46]	Oral	TiO_2_ NPs	6	Healthy	Rats	Dysbiosis, metabolic and colon changes, increased inflammation	Chronic metabolic effects are overlooked	No chronic metabolic assessment in cosmetics	Funding/Conflict of Interest—No
9. Mao et al. (2019) [47]	Oral	TiO_2_ NPs	4/group		Rats	Dysbiosis, hyperglycemia	Gestational diabetes risk	No pregnancy-specific guidance	Funding/Conflict of Interest—No
10. Shabbir etal. (2023) [48]	Oral	SIO_2_ NPs	5	Immunosuppressed	Mice	Microbiota shifts, no overt toxicity	Microbiome effects in susceptible hosts	No data for immunocompromised hosts	Funding—Yes Conflict of interest—No
11. Hirai et al. (2015) [49]	Topical	SiNPs agglomerates	5–12/group	Healthy	Mice	Increased IgE, anaphylaxis with allergen	Sensitisation risk	Allergy potentiation was not addressed	Funding/Conflict of Interest—No
12. Palmer et al. (2019) [50]	Topical	SiO_2_ NPs	NS	Healthy	Mice	Decreased inflammation in the contact dermatitis model	Therapeutic potential	Immunomodulation endpoints missing	Funding/Conflict of Interest—No

NS—Not stated in publication.

**Table 4:** Human vs. Animal Hazard Comparison according to types of nanoparticles.

Nanoparticle Type	Silver Nanoparticles (AgNPs)	Zinc Oxide (ZnO)	Silica Nanoparticles (SiNPs)	Titanium Dioxide (TiO_2_)
Number of studies	3 [43,44,45]	1 [40]	5 [39,41,48,49,50]	3 [42,46,47]
Regulatory relevance	No requirements for neurodevelopmental or microbiome testing in long-term cosmetic NP exposure.	Guidelines address topical safety but not chronic ingestion or ionic form comparisons.	No microbiome or allergen potentiation endpoints in leave-on cosmetic safety assessments.	No chronic metabolic or pregnancy-specific endpoints in SCCS/FDA guidelines.
Human evidence	Workers show chronic respiratory inflammation; dermal use in wound dressings → occasional argyria; microbiome effects in humans are not well studied.	Used widely in sunscreen; minimal systemic toxicity in healthy users; oral exposure data lacking.	Occupational exposure linked to chronic inflammation in inhalation settings; no consumer-use chronic dermal/oral studies.	Occupational exposure linked to respiratory inflammation; limited direct human gut/skin microbiome data; possible photocatalytic skin effects in sunscreen workers.
Animal Model	Gut microbiota shifts (↓ beneficial taxa), ↑ pro-inflammatory species, neurobehavioral changes in perinatal exposure, and immune modulation.	Haematologic, biochemical, and organ toxicity; ZnCl_2_ is more toxic than ZnO NPs.	Oral: microbiota/metabolite shifts without overt toxicity; Dermal: allergen co-exposure → ↑IgE/anaphylaxis; dermatitis model → reduced inflammation.	Gut dysbiosis, ↑ intestinal permeability, oxidative stress, metabolic changes (hyperglycemia, altered amino acids), and immune activation. Pregnancy exposure → gestational diabetes risk in rats.

Downregulation ↓, and Upregulation ↑.

**Table 5:** Country-Specific Regulatory Gap Table.

Jurisdiction	Main Regulatory Body	Current NP AssessmentScope	Gaps Relevant to Chronic Exposure & Microbiome
**European Union**	SCCS under EU Cosmetics Regulation (EC) No 1223/2009	Requires notification & safety assessment of NPs; acute &subchronic toxicity data; particle characterisation.	No requirement for chronic exposure testing; no microbiome or immune endpoint integration; pregnancy/vulnerable population data not mandated.
**United States**	FDA (Cosmetics not pre-approved, except colour additives)	Manufacturers are responsible for safety, voluntary registration, and toxicology data are rarely required unless safety concerns arise.	No NP-specific chronic exposure guidelines; no requirement for microbiome/barrier integrity studies; lack of occupational exposure considerations.
**Canada**	Health Canada (Cosmetic Regulations, Food and Drugs Act)	Ingredient Hotlist bans/restrictions; NP evaluation case-by-case; relies on existing toxicology.	No standardized NP chronic exposure framework; no gut–skin axis or microbiome endpoints.
**Japan**	MHLW under the Pharmaceutical and Medical Device Act	Cosmetics are regulated for safety; NP-specific risk assessment is rare; focus on product efficacy & safety at intended use.	No chronic NP exposure models; no immune or microbiome testing requirements
**Australia**	NICNAS (now AICIS)	NP definition included notification required for new industrial chemicals including NPs.	No specific chronic cosmetic-use testing for microbiome/immune outcomes; minimal dermal penetration guidance for NPs.

## Data Availability

All data used in this systematic review were extracted from publicly available peer-reviewed publications. The authors can provide extracted data tables upon reasonable request.

## References

[1] Honari G., Maibach H.I. (2014). Skin Structure and Function. Applied Dermatotoxicology: Clinical Aspects.

[2] Leprince C., Simon M. (2025). Epidermal lamellar bodies, essential organelles for the skin barrier. Front. Cell Dev. Biol..

[3] Clayton K., Vallejo A.F., Davies J., Sirvent S., Polak M.E. (2017). Langerhans Cells—Programmed by the Epidermis. Front. Immunol..

[4] Nguyen A.V., Soulika A.M. (2019). The Dynamics of the Skin’s Immune System. Int. J. Mol. Sci..

[5] Pat Y., Ogulur I., Yazici D., Mitamura Y., Cevhertas L., Küçükkase O.C., Mesisser S.S., Akdis M., Nadeau K., Akdis C.A. (2023). Effect of Altered Human Exposome on the Skin and Mucosal Epithelial Barrier Integrity. Tissue Barriers.

[6] Gu Y., Bian Q., Zhou Y., Huang Q., Gao J. (2022). Hair follicle-targeting drug delivery strategies for the management of hair follicle-associated disorders. Asian J. Pharm. Sci..

[7] Menichetti A., Mordini D., Montalti M. (2025). Penetration of Microplastics and Nanoparticles Through Skin: Effects of Size, Shape, and Surface Chemistry. J. Xenobiot..

[8] Zia S., Islam Aqib A., Muneer A., Fatima M., Atta K., Kausar T., Zaheer C.-N.F., Ahmad I., Saeed M., Shafique A. (2023). Insights into nanoparticles-induced neurotoxicity and cope up strategies. Front. Neurosci..

[9] Lim E.Y., Kim G.-D. (2024). Particulate Matter-Induced Emerging Health Effects Associated with Oxidative Stress and Inflammation. Antioxidants.

[10] Parrado C., Mercado-Saenz S., Perez-Davo A., Gilaberte Y., Gonzalez S., Juarranz A. (2019). Environmental Stressors on Skin Aging: Mechanistic Insights. Front. Pharmacol..

[11] Seweryn A. (2018). Interactions between Surfactants and the Skin—Theory and Practice. Adv. Colloid Interface Sci..

[12] Baur R., Kashon M., Lukomska E., Weatherly L.M., Shane H.L., Anderson S.E. (2023). Exposure to the Anti-Microbial Chemical Triclosan Disrupts Keratinocyte Function and Skin Integrity in a Model of Reconstructed Human Epidermis. J. Immunotoxicol..

[13] Lee C.-C., Lin Y.-H., Hou W.-C., Li M.-H., Chang J.-W. (2020). Exposure to ZnO/TiO_2_ Nanoparticles Affects Health Outcomes in Cosmetics Salesclerks. Int. J. Environ. Res. Public Health.

[14] Samberg M.E., Oldenburg S.J., Monteiro-Riviere N.A. (2010). Evaluation of Silver Nanoparticle Toxicity in Skin in Vivo and Keratinocytes in Vitro. Environ. Health Perspect..

[15] Knaggs H., Lephart E.D. (2023). Enhancing Skin Anti-Aging through Healthy Lifestyle Factors. Cosmetics.

[16] Jimenez-Sanchez M., Celiberto L.S., Yang H., Sham H.P., Vallance B.A. (2025). The gut–skin axis: A bi-directional, microbiota-driven relationship with therapeutic potential. Gut Microbes.

[17] Zhou Q., Verne G.N. (2018). Intestinal hyperpermeability: A gateway to multi-organ failure?. J. Clin. Investig..

[18] Du Y., He C., An Y., Huang Y., Zhang H., Fu W., Wang M., Shan Z., Xie J., Yang Y. (2024). The Role of Short Chain Fatty Acids in Inflammation and Body Health. Int. J. Mol. Sci..

[19] Prajaati S.K., Lekkala L., Yadav D., Jain S., Yadav H. (2025). Microbiome and Postbiotics in Skin Health. Biomedicines.

[20] Bertollo A.G., Santos C.F., Bagatini M.D., Ignácio Z.M. (2025). Hypothalamus-pituitary-adrenal and gut-brain axes in biological interaction pathway of the depression. Front. Neurosci..

[21] Xuan L., Ju Z., Skonieczna M., Zhou P.-K., Huang R. (2023). Nanoparticles-Induced Potential Toxicity on Human Health: Applications, Toxicity Mechanisms, and Evaluation Models. Med. Comm.

[22] U.S. Environmental Protection Agency (EPA) IRIS Glossary—Chronic Exposure. https://sor.epa.gov/sor_internet/registry/termreg/searchandretrieve/glossariesandkeywordlists/search.do?details=&vocabName=IRIS%20Glossary&filterTerm=chronic.

[23] Ghebretatios M., Schaly S., Prakash S. (2021). Nanoparticles in the Food Industry and Their Impact on Human Gut Microbiome and Diseases. Int. J. Mol. Sci..

[24] Aguwa C., Enwereji N., Santiago S., Hine A., Kels G.G., McGee J., Lu J. (2023). Targeting Dysbiosis in Psoriasis, Atopic Dermatitis, and Hidradenitis Suppurativa: The Gut–Skin Axis and Microbiome-Directed Therapy. Clin. Dermatol..

[25] Ramasamy M., Lee J. (2016). Recent Nanotechnology Approaches for Prevention and Treatment of Biofilm-Associated Infections on Medical Devices. BioMed Res. Int..

[26] Khanipour-Machiani M., Jamshidi S., Nikaein D., Khosravi A., Balal A. (2024). The Inhibitory Effects of Zinc Oxide Nanoparticles on Clinical Isolates of *Microsporumcanis* in Dogs and Cats. Vet. Med. Sci..

[27] SCCS (2023). Guidance on the Safety Assessment of Nanomaterials in Cosmetics.

[28] U.S. Food and Drug Administration (FDA) (2014). Guidance for Industry: Safety of Nanomaterials in Cosmetic Products.

[29] Rosa I., Marini M., Manetti M. (2021). Telocytes: An Emerging Component of Stem Cell Niche Microenvironment. J. Histochem. Cytochem..

[30] Manole C.G., Voiculescu V.M., Soare C., Ceafalan L.C., Gherghiceanu M., Hinescu M.E. (2024). Skin Telocytes Could Fundament the Cellular Mechanisms of Wound Healing in Platelet-Rich Plasma Administration. Cells.

[31] Ye Q., Yu Z.-H., Nie L., Wang F.-X., Mu G., Lu B. (2025). Understanding the Complex Role of Exosomes in Intestinal Ischemia–Reperfusion Injury: From Pathogenesis to Protection. Front. Pharmacol..

[32] Lima T.S.M., Souza W., Geaquinto L.R.O., Sanches P.L., Stępień E.L., Meneses J., Fernández-de Gortari E., Meisner-Kober N., Himly M., Granjeiro J.M. (2022). Nanomaterial Exposure, Extracellular Vesicle Biogenesis and Adverse Cellular Outcomes: A Scoping Review. Nanomaterials.

[33] Ma Y., Zhang J., Yu N., Shi J., Zhang Y., Chen Z., Jia G. (2023). Effect of Nanomaterials on Gut Microbiota. Toxics.

[34] Tang M., Zhang T., Xue Y., Wang S., Huang M., Yang Y., Lu M., Fan T., Li Y., Xu L. (2021). Do Engineered Nanomaterials Affect Immune Responses by Interacting with Gut Microbiota?. Front. Immunol..

[35] Mikiciuk J., Mikiciuk E., Wrońska A., Szterk A. (2016). Antimicrobial Potential of Commercial Silver Nanoparticles toward Probiotic Bacteria Isolated from Fermented Milk Products. J. Environ. Sci. Health Part B.

[36] De Pessemier B., Grine L., Debaere M., Maes A., Paetzold B., Callewaert C. (2021). Gut–Skin Axis: Current Knowledge of the Interrelationship between Microbial Dysbiosis and Skin Conditions. Microorganisms.

[37] Ni Q., Zhang P., Li Q., Han Z. (2022). Oxidative Stress and Gut Microbiome in Inflammatory Skin Diseases. Front. Cell Dev. Biol..

[38] Page M.J., McKenzie J.E., Bossuyt P.M., Boutron I., Hoffmann T.C., Mulrow C.D., Shamseer L., Tetzlaff J.M., Akl E.A., Brennan S.E. (2021). The PRISMA 2020 statement: An updated guideline for reporting systematic reviews. BMJ.

[39] Ryu H.J., Seong N.-W., So B.J., Seo H.-S., Kim J.-H., Hong J.-S., Park M.-K., Kim M.-S., Kim Y.-R., Cho K.-B. (2024). Evaluation of silica nanoparticle toxicity after topical exposure for 90 days. Int. J. Nanomed..

[40] Yazdanshenas M.R., Rezaei M.R., Kharkan J. (2025). Comparative toxicity of zinc oxide nanoparticles and zinc salts in male mice: Hematological, biochemical, and histopathological impacts. Toxicol. Rep..

[41] Landsiedel R., Hahn D., Ossig R., Ritz S., Sauer L., Buesen R., Rehm S., Wohlleben W., Groeters S., Strauss V. (2022). Gut microbiome and plasma metabolome changes in rats after oral gavage of nanoparticles: Sensitive indicators of possible adverse health effects. Part. Fibre Toxicol..

[42] Chen L., Guo Y., Hu C., Lam P.K.S., Lam J.C.W., Zhou B. (2018). Dysbiosis of gut microbiota by chronic coexposure to titanium dioxide nanoparticles and bisphenol A: Implications for host health in zebrafish. Environ. Pollut..

[43] Wilding L.A., Bassis C.M., Walacavage K., Hashway S., Leroueil P.R., Morishita M., Maynard A.D., Philbert M.A., Bergin I.L. (2016). Repeated dose (28 day) administration of silver nanoparticles of varied size and coating does not significantly alter the indigenous murine gut microbiome. Nanotoxicology.

[44] Van den Brule S., Ambroise J., Lecloux H., Levard C., Soulas R., De Temmerman P.-J., Palmai-Pallag M., Marbaix E., Lison D. (2016). Dietary silver nanoparticles can disturb the gut microbiota in mice. Part. Fibre Toxicol..

[45] Lyu Z., Ghoshdastidar S., Rekha K.R., Suresh D., Mao J., Bivens N., Kannan R., Joshi T., Rosenfeld C.S., Upendran A. (2021). Developmental Exposure to Silver Nanoparticles Leads to Long Term Gut Dysbiosis and Neurobehavioral Alterations. Sci. Rep..

[46] Chen Z., Han S., Zhou D., Zhou S., Jia G. (2019). Effects of Oral Exposure to Titanium Dioxide Nanoparticles on Gut Microbiota and Gut-Associated Metabolism in Vivo. Nanoscale.

[47] Mao Z., Li Y., Dong T., Zhang L., Zhang Y., Li S., Hu H., Sun C., Xia Y. (2019). Exposure to Titanium Dioxide Nanoparticles During Pregnancy Changed Maternal Gut Microbiota and Increased Blood Glucose of Rat. Nanoscale Res. Lett..

[48] Shabbir S., Hu Y., He X., Huang K., Xu W. (2023). Toxicity and Impact of Silica Nanoparticles on the Configuration of Gut Microbiota in Immunodeficient Mice. Microorganisms.

[49] Hirai T., Yoshioka Y., Takahashi H., Ichihashi K., Udaka A., Mori T., Nishijima N., Yoshida T., Nagano K., Kamada H. (2015). Cutaneous Exposure to Agglomerates of Silica Nanoparticles and Allergen Results in IgE-Biased Immune Response and Increased Sensitivity to Anaphylaxis in Mice. Part. Fibre Toxicol..

[50] Palmer B.C., Jatana S., Phelan-Dickinson S.J., DeLouise L.A. (2019). Amorphous Silicon Dioxide Nanoparticles Modulate Immune Responses in a Model of Allergic Contact Dermatitis. Sci. Rep..

[51] Lorenz C., von Goetz N., Scheringer M., Wormuth M., Hungerbühler K. (2011). Potential Exposure of German Consumers to Engineered Nanoparticles in Cosmetics and Personal Care Products. Nanotoxicology.

[52] Mondéjar-López M., López-Jiménez A.J., Abad-Jordá M., Rubio-Moraga A., Ahrazem O., Gómez-Gómez L., Niza E. (2021). Biogenic Silver Nanoparticles from *Iris tuberosa* as Potential Preservative in Cosmetic Products. Molecules.

[53] Hosny A.E.-D.M.S., Kashef M.T., Taher H.A., El-Bazza Z.E. (2017). The Use of Unirradiated and γ-Irradiated Zinc Oxide Nanoparticles as a Preservative in Cosmetic Preparations. Int. J. Nanomed..

[54] Nigam P.K. (2009). Adverse Reactions to Cosmetics and Methods of Testing. Indian J. Dermatol. Venereol. Leprol..

[55] Skibska A., Perlikowska R. (2021). Signal Peptides—Promising Ingredients in Cosmetics. Curr. Protein Pept. Sci..

[56] Balwierz R., Biernat P., Jasińska-Balwierz A., Siodłak D., Kusakiewicz-Dawid A., Kurek-Górecka A., Olczyk P., Ochędzan-Siodłak W. (2023). Potential Carcinogens in Makeup Cosmetics. Int. J. Environ. Res. Public Health.

[57] Roso A., Aubert A., Cambos S., Vial F., Schäfer J., Belin M., Gabriel D., Bize C. (2024). Contribution of Cosmetic Ingredients and Skin Care Textures to Emotions. Int. J. Cosmet. Sci..

[58] Bhat B.B., Kamath P.P., Chatterjee S., Bhattacherjee R., Nayak U.Y. (2022). Recent updates on nanocosmeceutical skin care and anti-aging products. Curr. Pharm. Des..

[59] Liu L. (2020). Penetration of Surfactants into Skin. J. Cosmet. Sci..

[60] Yin C., Yu L., Feng L., Zhou J.T., Du C., Shao X., Cheng Y. (2024). Nanotoxicity of two-dimensional nanomaterials on human skin and the structural evolution of keratin protein. Nanotechnology.

[61] Nohynek G.J., Lademann J., Ribaud C., Roberts M.S. (2007). Grey goo on the skin? Nanotechnology, cosmetic and sunscreen safety. Crit. Rev. Toxicol..

[62] Eroğlu C., Sinani G., Ülker Z. (2023). Current state of lipid nanoparticles (SLN and NLC) for skin applications. Curr. Pharm. Des..

[63] Namdar R., Nafisi S. (2018). Nanodiamond Applications in Skin Preparations. Drug Discov. Today.

[64] Puglia C., Santonocito D. (2019). Cosmeceuticals: Nanotechnology-Based Strategies for the Delivery of Phytocompounds. Curr. Pharm. Des..

[65] Matsuo K., Hirobe S., Okada N., Nakagawa S. (2016). Analysis of Skin Permeability and Toxicological Properties of Amorphous Silica Particles. Biol. Pharm. Bull..

[66] Busch L., Keziban Y., Dähne L., Keck C.M., Meinke M.C., Lademann J., Patzelt A. (2021). The impact of skin massage frequency on the intrafollicular transport of silica nanoparticles: Validation of the ratchet effect on an ex vivo porcine skin model. Eur. J. Pharm. Biopharm..

[67] Kaur J., Anwer M.K., Sartaj A., Panda B.P., Ali A., Zafar A., Kumar V., Gilani S.J., Kala C., Taleuzzaman M. (2022). ZnO nanoparticles of *Rubia cordifolia* extract formulation developed and optimized with QbD application, considering ex vivo skin permeation, antimicrobial and antioxidant properties. Molecules.

[68] Sheng W., Seare W.J., DiBernardo B., Alhasan A.H., Cory E., Chasan P., Sah R.L., Almutairi K.M., Almutairi A. (2018). A single-blind study evaluating the efficacy of gold nanoparticle photothermal-assisted liposuction in an ex vivo human tissue model. Aesthet. Surg. J..

[69] Mousavisani S.Z., Raoof J.-B., Cheung K.Y., Hernández Camargo A.R., Ruzgas T., Turner A.P.F., Mak W.C. (2019). Integrating an ex-vivo skin biointerface with electrochemical DNA biosensor for direct measurement of the protective effect of UV blocking agents. Biosens. Bioelectron..

[70] Ang She Tou K., Rehman K., Wan Ishak W.M., Zulfakar M.H. (2019). Influence of omega fatty acids on skin permeation of a coenzyme Q10 nanoemulsion cream formulation: Characterization, in silico and ex vivo determination. Drug Dev. Ind. Pharm..

[71] Demir E., Turna Demir F., Marcos R., Louro H., Silva M.J. (2022). Drosophila as a Suitable In Vivo Model in the Safety Assessment of Nanomaterials. Nanotoxicology in Safety Assessment of Nanomaterials.

[72] Gonzalez-Moragas L., Berto P., Vilches C., Quidant R., Kolovou A., Santarella-Mellwig R., Schwab Y., Stürzenbaum S., Roig A., Laromaine A. (2017). In vivo testing of gold nanoparticles using the *Caenorhabditis elegans* model organism. Acta Biomater..

[73] Fröhlich E. (2015). Value of phagocyte function screening for immunotoxicity of nanoparticles in vivo. Int. J. Nanomed..

[74] Durbakula K., Prabhu V., Jose M. (2017). Genotoxicity of non-alcoholic mouth rinses: A micronucleus and nuclear abnormalities study with fluorescent microscopy. J. Investig. Clin. Dent..

[75] Liu W., Jie L., Liu D., Makino E.T., Krutmann J., Mehta R.C. (2023). Protective effects of a day/night dual-antioxidant serum on skin: A randomized, regimen-controlled study in Chinese women exposed to air pollution. J. Cosmet. Dermatol..

[76] Oh S., Jeong J., Kim M., Jin X., Zheng S., Kim Y.-M., Yi T.-H. (2024). A study of anti-wrinkle functions and improvement of cream with *Phaseolus angularis*. Int. J. Cosmet. Sci..

[77] Prakoeswa C.R.S., Huda B.K.N., Indrawati D., Umborowati M.A., Anggraeni S., Damayanti, Murtiastutik D., Kerob D. (2025). Effectiveness and tolerability of an emollient “plus” compared to urea 10% in patients with mild-to-moderate atopic dermatitis. J. Cosmet. Dermatol..

[78] Souza C., de Freitas L.A.P., Maia Campos P.M.B.G. (2017). Topical Formulation Containing Beeswax-Based Nanoparticles Improved In Vivo Skin Barrier Function. AAPS PharmSciTech.

[79] Kurtz C.C., Mitchell S., Nielsen K., Crawford K.D., Mueller-Spitz S.R. (2020). Acute high-dose titanium dioxide nanoparticle exposure alters gastrointestinal homeostasis in mice. J. Appl. Toxicol..

[80] Annangi B., Rubio L., Alaraby M., Bach J., Marcos R., Hernández A. (2016). Acute and long-term in vitro effects of zinc oxide nanoparticles. Arch. Toxicol..

[81] Mangalampalli B., Dumala N., Grover P. (2017). Acute oral toxicity study of magnesium oxide nanoparticles and microparticles in female albino Wistar rats. Regul. Toxicol. Pharmacol..

[82] Nelson M.A., Domann F.E., Bowden G.T., Hooser S.B., Fernando Q., Carter D.E. (1993). Effects of acute and subchronic exposure of topically applied fullerene extracts on the mouse skin. Toxicol. Ind. Health.

[83] Korani M., Rezayat S.M., Gilani K., Arbabi Bidgoli S., Adeli S. (2011). Acute and subchronic dermal toxicity of nanosilver in guinea pig. Int. J. Nanomed..

[84] Hansen T., Tillmann T., Wiench K., Creutzenberg O. (2025). Studies on acute dermal toxicity and dermal absorption of a nanoform zinc oxide (ZnO; NM-111) in rats. Toxicol. Lett..

[85] Yuan Z., Yan R., Fu Z., Wu T., Ren C. (2024). Impact of physicochemical properties on biological effects of lipid nanoparticles: Are they completely safe. Sci. Total Environ..

[86] Schmid G., Kreyling W.G., Simon U. (2017). Toxic Effects and Biodistribution of Ultrasmall Gold Nanoparticles. Arch. Toxicol..

[87] Cazenave J., Ale A., Bacchetta C., Rossi A.S. (2019). Nanoparticles toxicity in fish models. Curr. Pharm. Des..

[88] Severino P., Silveira E.F., Loureiro K., Chaud M.V., Antonini D., Lancellotti M., Sarmento V.H., da Silva C.F., Santana M.H.A., Souto E.B. (2017). Antimicrobial activity of polymyxin-loaded solid lipid nanoparticles (PLX-SLN): Characterization of physicochemical properties and in vitro efficacy. Eur. J. Pharm. Sci..

[89] Utembe W., Tlotleng N., Kamng’ona A.W. (2022). A systematic review on the effects of nanomaterials on gut microbiota. Curr. Res. Microbiol. Sci..

[90] Rinninella E., Cintoni M., Raoul P., Mora V., Gasbarrini A., Mele M.C. (2021). Impact of Food Additive Titanium Dioxide on Gut Microbiota Composition, Microbiota-Associated Functions, and Gut Barrier: A Systematic Review of In Vivo Animal Studies. Int. J. Environ. Res. Public Health.

[91] Bettini S., Boutet-Robinet E., Cartier C., Coméra C., Gaultier E., Dupuy J., Naud N., Taché S., Grysan P., Reguer S. (2017). Food-Grade TiO_2_ Impairs Intestinal and Systemic Immune Homeostasis, Initiates Preneoplastic Lesions and Promotes Aberrant Crypt Development in the Rat Colon. Sci. Rep..

[92] Mohammed Y.H., Holmes A., Haridass I.N., Sanchez W.Y., Studier H., Grice J.E., Benson H.A.E., Roberts M.S. (2019). Support for the Safe Use of Zinc Oxide Nanoparticle Sunscreens: Lack of Skin Penetration or Cellular Toxicity after Repeated Application in Volunteers. J. Investig. Dermatol..

[93] Khabir Z., Holmes A.M., Lai Y.J., Liang L., Deva A., Polikarpov M.A., Roberts M.S., Zvyagin A.V. (2021). Human Epidermal Zinc Concentrations after Topical Application of ZnO Nanoparticles in Sunscreens. Int. J. Mol. Sci..

[94] Babaei V., Ashtarinezhad A., Torshabi M., Teimourian S., Shahmirzaie M., Abolghasemi J., ZeraatgarGohardani H., Vernousfaderani E.K., Shirazi F.H. (2024). High Inflammatory Cytokines Gene Expression Can Be Detected in Workers with Prolonged Exposure to Silver and Silica Nanoparticles in Industries. Sci. Rep..

[95] Ratanapokasatit Y., Laisuan W., Rattananukrom T., Petchlorlian A., Thaipisuttikul I., Sompornrattanaphan M. (2022). How Microbiomes Affect Skin Aging: The Updated Evidence and Current Perspectives. Life.

[96] Naik S., Bouladoux N., Wilhelm C., Molloy M.J., Salcedo R., Kastenmuller W., Deming C., Quinones M., Koo L., Conlan S. (2012). Compartmentalized Control of Skin Immunity by Resident Commensals. Science.

[97] Henkler F., Tralau T., Tentschert J., Kneuer C., Haase A., Platzek T., Luch A., Götz M.E. (2012). Risk Assessment of Nanomaterials in Cosmetics: A European Union Perspective. Arch. Toxicol..

[98] Yoshioka Y., Kuroda E., Hirai T., Tsutsumi Y., Ishii K.J. (2017). Allergic Responses Induced by the Immunomodulatory Effects of Nanomaterials upon Skin Exposure. Front. Immunol..

[99] Allan J., Belz S., Hoeveler A., Hugas M., Okuda H., Patri A., Rauscher H., Silva P., Slikker W., Sokull-Kluettgen B. (2021). Regulatory Landscape of Nanotechnology and Nanoplastics from a Global Perspective. Regul. Toxicol. Pharmacol..

[100] Weir A., Westerhoff P., Fabricius L., Hristovski K., von Goetz N. (2012). Titanium Dioxide Nanoparticles in Food and Personal Care Products. Environ. Sci. Technol..

[101] Saweres-Argüelles C., Ramírez-Novillo I., Vergara-Barberán M., Carrasco-Correa E.J., Lerma-García M.J., Simó-Alfonso E.F. (2023). Skin Absorption of Inorganic Nanoparticles and Their Toxicity: A Review. Eur. J. Pharm. Biopharm..

[102] Pinget G., Tan J., Janac B., Kaakoush N.O., Angelatos A.S., O’Sullivan J., Koay Y.C., Sierro F., Davis J., Divakarla S.K. (2019). Impact of the Food Additive Titanium Dioxide (E171) on Gut Microbiota–Host Interaction. Front. Nutr..

[103] Cao X., Han Y., Gu M., Du H., Song M., Zhu X., Ma G., Pan C., Wang W., Zhao E. (2020). Foodborne Titanium Dioxide Nanoparticles Induce Stronger Adverse Effects in Obese Mice than Non-Obese Mice: Gut Microbiota Dysbiosis, Colonic Inflammation, and Proteome Alterations. Small.

[104] Ruiz P.A., Morón B., Becker H.M., Lang S., Atrott K., Spalinger M.R., Scharl M., Wojtal K.A., Fischbeck-Terhalle A., Frey-Wagner I. (2017). Titanium Dioxide Nanoparticles Exacerbate DSS-Induced Colitis: Role of the NLRP3 Inflammasome. Gut.

[105] Ma Y., Yu N., Lu H., Shi J., Zhang Y., Chen Z., Jia G. (2023). Titanium Dioxide Nanoparticles: Revealing the Mechanisms Underlying Hepatotoxicity and Effects in the Gut Microbiota. Arch. Toxicol..

[106] Chen Z., Zhou D., Han S., Zhou S., Jia G. (2019). Hepatotoxicity and the Role of the Gut–Liver Axis in Rats after Oral Administration of Titanium Dioxide Nanoparticles. Part FibreToxicol..

[107] Agans R.T., Gordon A., Hussain S., Paliy O. (2019). Titanium Dioxide Nanoparticles Elicit Lower Direct Inhibitory Effect on Human Gut Microbiota Than Silver Nanoparticles. Toxicol. Sci..

[108] Wang S., Ilves M., Mäenpää K., Zhao L., El-Nezami H., Karisola P., Alenius H. (2024). ZnO Nanoparticles as Potent Inducers of Dermal Immunosuppression in Contact Hypersensitivity in Mice. ACS Nano.

[109] Júnior D.M., Hausen M.A., Asami J., Higa A.M., Leite F.L., Mambrini G.P., Rossi A.L., Komatsu D., Duek E.A.R. (2021). A New Dermal Substitute Containing Polyvinyl Alcohol with Silver Nanoparticles and Collagen with Hyaluronic Acid: In Vitro and In Vivo Approaches. Antibiotics.

[110] Mukhopadhyay S., Youssef S.H., Song Y., Nayak U.Y., Garg S. (2025). Harnessing the Power of Antimicrobial Peptides: From Mechanisms to Delivery Optimization for Topical Infections. Antibiotics.

[111] Najahi-Missaoui W., Arnold R.D., Cummings B.S. (2020). Safe Nanoparticles: Are We There Yet?. Int. J. Mol. Sci..

[112] Duan S., Wang H., Gao Y., Wang X., Lyu L., Wang Y. (2023). Oral Intake of Titanium Dioxide Nanoparticles Affect the Course and Prognosis of Ulcerative Colitis in Mice: Involvement of the ROS-TXNIP-NLRP3 Inflammasome Pathway. Part. Fibre Toxicol..

[113] Meier M.J., Nguyen K.C., Crosthwait J., Kawata A., Rigden M., Leingartner K., Wong A., Holloway A., Shwed P.S., Beaudette L. (2021). Low Dose Antibiotic Ingestion Potentiates Systemic and Microbiome Changes Induced by Silver Nanoparticles. NanoImpact.

[114] Wu Y., Cao X., Du H., Guo X., Han Y., McClements D.J., Decker E., Xing B., Xiao H. (2023). Adverse Effects of Titanium Dioxide Nanoparticles on Beneficial Gut Bacteria and Host Health Based on Untargeted Metabolomics Analysis. Environ. Res..

[115] Larese Filon F., Bello D., Cherrie J.W., Sleeuwenhoek A., Spaan S., Brouwer D.H. (2016). Occupational Dermal Exposure to Nanoparticles and Nano-Enabled Products: Part I—Factors Affecting Skin Absorption. Int. J. Hyg. Environ. Health.

[116] Brouwer D.H., Spaan S., Roff M., Sleeuwenhoek A., Tuinman I., Goede H., van Duuren-Stuurman B., Filon F.L., Bello D., Cherrie J.W. (2016). Occupational Dermal Exposure to Nanoparticles and Nano-Enabled Products: Part 2, Exploration of Exposure Processes and Methods of Assessment. Int. J. Hyg. Environ. Health.

[117] Hartwig O., Loretz B., Nougarede A., Jary D., Sulpice E., Gidrol X., Navarro F., Lehr C.M. (2022). Leaky Gut Model of the Human Intestinal Mucosa for Testing siRNA-Based Nanomedicine Targeting JAK1. J. Control. Release.

[118] Li J., Tang M., Xue Y. (2019). Review of the Effects of Silver Nanoparticle Exposure on Gut Bacteria. J. Appl. Toxicol..

[119] Akombaetwa N., Ilangala A.B., Thom L., Memvanga P.B., Witika B.A., Buya A.B. (2023). Current Advances in Lipid Nanosystems Intended for Topical and Transdermal Drug Delivery Applications. Pharmaceutics.

[120] Aljuffali I.A., Huang C.H., Fang J.Y. (2015). Nanomedical Strategies for Targeting Skin Microbiomes. Curr. Drug Metab..

[121] Jain A., Jain P., Kurmi J., Jain D., Jain R., Chandel S., Sahu A., Mody N., Upadhaya S., Jain A. (2014). Novel Strategies for Effective Transdermal Drug Delivery: A Review. Crit. Rev. Ther. Drug Carr. Syst..

[122] Akdis C.A. (2021). Does the Epithelial Barrier Hypothesis Explain the Increase in Allergy, Autoimmunity and Other Chronic Conditions?. Nat. Rev. Immunol..

[123] Celebi Sozener Z., Özbey Yücel Ü., Altiner S., OzdelOztürk B., Cerci P., Türk M., Gorgülü Akin B., Akdis M., Yilmaz I., Ozdemir C. (2022). The External Exposome and Allergies: From the Perspective of the Epithelial Barrier Hypothesis. Front. Allergy.

[124] Honda K., Littman D.R. (2016). The Microbiota in Adaptive Immune Homeostasis and Disease. Nature.

[125] Zhou G., Yu R., Ahmed T., Jiang H., Zhang M., Lv L., Alhumaydhi F.A., Allemailem K.S., Li B. (2021). Biosynthesis and Characterization of Zinc Oxide Nanoparticles and Their Impact on the Composition of Gut Microbiota in Healthy and Attention-Deficit Hyperactivity Disorder Children. Front. Microbiol..

